# *In vitro* study comparing sealing ability of resin-based and bioceramic sealers using single-cone obturation technique

**DOI:** 10.6026/973206300213436

**Published:** 2025-10-31

**Authors:** Nimiya V.H., Vishnu Chernad, Rekha P. Thankachan, Anoop Samuel, Ranjith K., Elvis Chiramel David

**Affiliations:** 1 Department of Conservative Dentistry and Endodontics, MES Dental College, Perinthalmanna, Malappuram, Kerala, India; 2 Private Dental Practitioner, Malappuram, Kerala, India

**Keywords:** Bioceramic sealers, optical microscope, resin-based sealer, sealing ability, single-cone obturation technique

## Abstract

This *in vitro* study was performed to compare the sealing ability of different bioceramic sealers- CeraSeal, DiaRoot BC and BioCera BC and that of AH plus sealer (n=17/group), utilizing single-rooted mandibular premolars. The specimens underwent examination under a stereomicroscope (magnification x2-20) for evaluation of dye penetration. Thus, we show that Ceraseal BC exhibited the highest sealing potential, followed by AH Plus, DiaRoot and Biocera BC.

## Background:

A good quality obturation will effectively fence off the apex of root canal (RC) and would avert any further entry of the microorganisms and will provide a hermetic seal to all 'portals of exit', thus avoids any chances of misfortunes like periapical infection or inflammatory changes. The core filling material universally is gutta-percha and hence not many arguments in that category due to its inertness, plasticity and solvent solubility [1]. It provides felicitous filling in a relatively tapered RC but the irregularities in the canal anatomy would leave of voids that could burgeon bacterial growth. And the bacterial ingress through these voids could cause failure of RC treatment [2]. Hence, there comes the role of root canal sealers (RCS). Many sealers are available nowadays with different qualities and properties. Each type has its own limitations and the quest for the ideal sealer continues. The composition-based classification of these sealers has a very broad category of Bioceramic sealers (BCS) and resin-based sealers (RBS). Of which, the RBS are very well studied and documented and one of the most famous sealers in that category is AH plus sealer. It became famous owing to its ease to handle, excellent biocompatibility, high radio-opacity and ease of availability. The sealer AH Plus comes under the family of epoxy-bis-phenol resin, which also possesses adamantine and bonds well to the RC [3]. The RBS are famous for their sealing ability, cohesion and adhesion and hence contribute to the monoblock effect. Methacrylate adhesive coated gutta-percha would have a moisture-friendly portion, which could be bonded with methacrylate resin and a hydrophobic portion, which would pair with the polyisoprene substrate of gutta-percha would resulting in a healthy bond between gutta-percha and resin adhesive, ending up in the formation of the RC monoblock, which would eventually help to attain a total seal of the canal space. However, it has got many drawbacks such as a faster setting time, tendency to shrink away from the canal walls, resulting in premature debonding from the RC wall [4]. However, AH Plus sealer is very unique in its sealing ability and hardly any sealers could match that particular quality of AH Plus sealer only until the bioceramic sealers came in to the market, storming with its unparalleled biocompatibility and the ever-sought property of bioactivity that it is arguably said to possess. The unique chemical composition and crystalline structure of BCS are similar to those of tooth and apatite materials, thereby enhancing the adhesion between the RCS and the radicular dentine. Additionally, the RCS should discourage bacterial growth and be resistant to dissolution and the BCS is one such sealer [5]. The BCS, being biocompatible and hydrophilic, expands upon setting, forming a 'self-seal.' This expansion can reach up to 0.2% upon completion of the setting reaction [6]. This expansion, along with chemical and micromechanical bonding, enhances the sealer's adhesion to the RC walls. Additionally, the high pH (12.8) during the initial 24 hours of the setting process makes the RCS strongly antibacterial. It also exhibits excellent tissue-regeneration capability.

Among the Calcium silicate Bioceramic sealers, the most popular one arguably is CeraSeal (META BIOMED, Cheongju, Korea) since CeraSeal offers an antimicrobial action and is highly biocompatible owing to its elevated pH of 12.73. It also demonstrates excellent radiopacity (8 mm Al), provides good sealing ability and is suitable for use with single cone sealer-based obturation. This technique utilizes a single gutta-percha cone, with the sealer thickness inside the canal primarily dependent on how well the cone adapts to the RC walls [7]. Single-cone obturation has gained popularity among practitioners because it allows for faster completion of root canal treatments (RCT) with effective results. In addition to CeraSeal, other newer bioceramic sealers, such as BioCera BC Sealer and DiaRoot BC, were included in the comparison for this study. BioCera BC is a premixed paste bioceramic sealer, while DiaRoot BC is a newly launched product, with limited available literature focusing on its bond strength and physical properties. Despite the growing use of these newer Bioceramic sealers, there remains a lack of comprehensive studies comparing their sealing properties with traditional resin-based sealers and other bioceramic systems. Henceforth, this study aims to fill this gap by directly comparing the sealing abilities of these newer bioceramic sealers with AH Plus. One of the popular techniques in measuring the sealing potential of several obturation materials and techniques involves direct calibration of dye penetration. In this study, methylene blue dye was selected because its molecular size is comparable to that of bacterial by-products like butyric acid, which could potentially leach out from infected RC and irritate periapical tissues. Additionally, methylene blue is simple to handle and its ease of altering the pH and availability further enhance its advantages [8]. Even though there are many properties, the bioceramic sealers are now a frontrunner in root canal treatment and undoubtedly have an edge over the resin-based sealers. Hence, this *in-vitro* research was performed to contrast the sealing potential of different bioceramic sealers - CeraSeal, DiaRoot BC and BioCera BC and that of AH plus sealer. Therefore, it is of interest to study of the sealing ability of different bioceramic sealers and a resin-based sealer with single-cone obturation technique.

## Materials and Methods:

The *in-vitro* investigation was carried out in the Department of Conservative Dentistry and Endodontics, MES Dental College, Perinthalmanna, Department of Microbiology, MES Medical College, Perinthalmanna and National Institute of Technology, Calicut. The study was conducted between 1st June 2023 and 31st December 2024. The extracted human mandibular premolars. The sample size was determined using the formula: (see PDF)

Where Z α/2 = 1.96; Z β =1.28(Power of the study =90%); SD =Standard deviation = 0.27; d1-d2 = mean difference = 0.31 [9]. The estimated minimum sample size was 17 per group. Thus, the overall sample size was 85. The study included single-rooted mandibular premolars extracted for orthodontic reasons, teeth with mature apices and premolars without dental caries. The teeth with resorptive defects, root caries, or root fracture, acute canal curvature and calcifications, developmental anomalies and open apex. 85 human extracted single-rooted intact mandibular premolars with closed apices and straight canals without any acute visible curvature were selected to confirm that this digital radiograph was taken. After removal of all the debris teeth were placed in 2% sodium hypochlorite (Septodent, Saint Maur des Fossés, France) for 2 hrs and then placed in normal saline. 12mm from the apex tooth was decoronated and canals were accessed, instrumentation was done up to 11mm working length by use of Protaper Universal (Dentsply, Maillefer, Ballaigues, Switzerland) rotary file employing Schilder's technique as a base. RCs were periodically irrigated with freshly prepared 5.25% sodium hypochlorite (CERKAMED CHLORAXID) and 17%EDTA solution (PREVEST Denpro Ltd) alternatively between instrumentation and the final irrigant used was normal saline. Sterile paper points have been utilised for drying the RCs. Single cone technique of obturation was performed using the corresponding size (F2) as the master cone (Dentsply, Maillefer, Ballaigues, Switzerland).

The specimens were randomly grouped into five of 17 samples each. Group 1: GP utilizing CERASEAL BCS (META BIOMED). Group 2: GP utilizing DIAROOT BCS (DIADENT, South Korea). Group 3: GP using BIOCERA BCS (NINETEN NT). Group 4: GP using AH plus RCS (DENTSPLY, Maillefer, Ballaigues, Switzerland). The single cone obturation technique was used in all the four groups. Group 5: negative control group-gutta-percha alone is used (no sealer). Following obturation of every group of samples, coronally 2mm of GP is removed with a heated instrument after the sealer had hardened and the coronal opening was restored with GIC (FUJI TYPE IX) and placed in an incubator at 100% humidity at 37°c for 48h to enable final setting of sealer. Except for negative control, all the radicular surfaces of samples were then cleaned; dried and double coats of nail varnish with each and every coating were methodically applied, excluding the apical 2mm and given sufficient time to dry before successive applications were administered. Samples were put up in dye- methylene blue 1% in a transparent non-reactive container like glass for 72hr at 37°C in an incubator. Samples were made to stand erect employing a sticky wax, letting the dye enter in and go upwards due to capillary action. Once the tooth is taken out from the dye, it is cleansed under running tap water and the nail varnish removal was done using a bard-parker blade. Teeth were then subjected to the process of demineralization which was done by keeping them in a 5% nitric acid solution and in-order to keep the concentration, it was replenished daily for 5 days. The next step is dehydration, which was performed using 70%, 80%, 90% alcohol, in which the teeth were kept for 1h in each concentration and finally the cleaning process was accomplished by immersing in methyl salicylate solution. The samples were sectioned longitudinally in a path roughly parallel to the long axis of the tooth. Specimens were then examined under a stereomicroscope (magnification x2-20) for evaluation of dye penetration. The measurement of dye penetration was documented in millimetres.

## Results:

The sealing potential of four different sealers (Ceraseal BC, Diaroot BC, Biocera BC and AH plus) were determined based on their dye penetration values, where lower values indicate better sealing ability. Ceraseal BC demonstrated the best sealing ability with the lowest mean dye penetration value and the most consistent performance. In contrast, Biocera BC had the least sealing ability, with the highest mean dye penetration value. AH plus and Diaroot BC fell between these two extremes, with AH plus showing slightly better performance than Diaroot BC. The given ANOVA results compare the mean dye penetration values of the four sealers (Ceraseal BC, Diaroot BC, Biocera BC and AH Plus) to determine whether there are significant variances in their sealing abilities. The results of the ANOVA test points out that, there is a statistically significant difference in the sealing potential (as measured by dye penetration values) among the four sealers (Ceraseal BC, Diaroot BC, Biocera BC and AH Plus) (F-statistic = 36.520, p-value = 0.000) (Table 1 - see PDF). The multiple comparisons table summarizes the pairwise differences between the mean dye penetration values of the four sealers. It identifies which sealers have statistically significant differences in their sealing ability. Ceraseal BCS has the best sealing potential, significantly better than Diaroot BCS and Biocera BCS but similar to AH plus. Biocera BCS has the least sealing ability, significantly lower than all other sealers. AH plus performs better than Diaroot BCS and Biocera BCS and similarly to Ceraseal BCS. Diaroot BCS has intermediate sealing ability, better than Biocera BCS but worse than Ceraseal BCS and AH plus. The analysis confirms that Ceraseal BCS is the most effective sealer for preventing dye penetration, followed closely by AH plus. Biocera BCS is the least effective, with the highest mean dye penetration value (Table 2 - see PDF).

## Discussion:

Achieving a hermetic and three-dimensional seal is a cornerstone of the success of RC treatment. A secure seal of the treated RC is crucial for preventing the re-entry of bacteria, which could otherwise lead to reinfection and treatment failure. AH Plus, while exhibiting slightly higher dye penetration, still performed well, as multiple comparisons showed no statistically significant difference between AH Plus and CeraSeal BCS. These findings indicate that both Ceraseal BCS and AH Plus perform comparably in terms of sealing ability, but Ceraseal BC appears to have a slight edge in reducing microleakage. AH Plus, an RBS, has long been regarded as the benchmark standard due to its extensive body of research supporting its sealing properties [10, 11]. Given the growing interest in bioceramic materials, which offer distinct advantages such as biocompatibility and bioactivity, this study aimed to evaluate the sealing abilities of newer BCS, including Ceraseal BCS, Diaroot BCS and Biocera BCS, in comparison to AH Plus. The primary mechanism involves tricalcium silicate and dicalcium silicate particles, which, when exposed to humidity, form Ca2+ silicate and Ca2+ hydroxide needles in the hydrate phase. These particles slowly release Ca2+ and hydroxyl (OH-) ions when in contact with phosphate-containing fluids, enabling the precipitation of Ca2+-deficient apatite through the formation of an initial amorphous Ca2+ phosphate. Furthermore, the Ca2+ silicate hydrates release OH- and Ca2+ ions when interacting with human fluids that contain phosphates, leading to hydroxyapatite formation [12]. Mandibular premolars with single RCs were selected in this study due to their oval-shaped canal morphology. This shape tends to create a gap between the rounded gutta-percha and the RC walls, which requires a significant amount of sealer to fill effectively. Ensuring a proper seal is vital for preventing potential leakage, which could compromise the success of the RC treatment [13]. To ensure standardization and reduce variability in the preparation, all teeth were decoronated and their root lengths were adjusted to a uniform 12 mm [14]. This precise root length was carefully selected to ensure consistency across the study, providing a solid baseline for comparison. Additionally, RCs were shaped using Protaper Universal rotary files till F2 (Dentsply, Maillefer, Ballaigues, Switzerland), following Schilder's technique [15]. This technique focuses on achieving a continuous taper in the canals, optimizing the preparation for optimal sealing and reducing variability in canal morphology. This standardized approach to canal shaping was critical to ensure consistency across the specimens, minimizing factors that could influence the outcomes of the sealing tests. The Olympus BX51 Microscope (Olympus) with magnification ranging from 50 to 1000x was employed to observe and measure dye penetration. This microscope's advanced imaging capabilities, including brightfield, darkfield and phase contrast, allowed for detailed visualization of the dye penetration process and enhanced the accuracy of the analysis. To obtain precise longitudinal sections of the roots, Baincut LSS metallographic equipment was used. This slow-speed cutting tool ensured controlled and accurate sectioning, maintaining the integrity of the tooth structure for subsequent evaluation [16]. The sealing potential of RCS was assessed using various micro-leakage tests and *in-vitro* methods, including dye penetration tests, immersion tests and sectional analysis techniques. These tests were complemented by advanced imaging techniques such as scanning electron microscopy (SEM) and confocal laser scanning microscopy (CLSM), which provided detailed knowledge into the surface morphology and structural integrity of the seal. Other diagnostic tools, like bacterial microleakage test, micro-CT and nano-CT, are also commonly used to assess the extent of leakage and the ability of the sealer to prevent bacterial ingress [17]. In this study, methylene blue dye was selected for its high sensitivity, which allows for the detection of even minute leakage. Its particle size is comparable to that of microorganisms and their metabolites, making it an ideal indicator for assessing microleakage and potential bacterial penetration within the sealed root canal system [18, 19]. Ceraseal BCS is a pre-mixed BCS containing bioactive components such as 20-30% tricalcium silicate, 1-10% dicalcium silicate, 1-10% tricalcium aluminate and 45-50% zirconium dioxide as radiopacifiers [20].

In comparison, AH Plus is a resin-based sealer known for its satisfactory sealing properties, minimal dissolution in the oral environment and lack of bioactivity. It features a paste and paste system and is provided in two separate tubes within a double-barreled syringe. The composition of AH Plus includes silicone oils and other ingredients, with a thin film having an approximate thickness of 25 microns, well below the ISO standard requirement of less than 50 microns for RC sealing materials [21]. DiaRoot BCS, a newer BCS, has yet to be studied extensively for its physical properties [22]. Its composition includes dicalcium silicate, tricalcium silicate, calcium phosphate monobasic, tantalum pentoxide and amorphous silicon oxide. While its composition is similar to that of mineral trioxide aggregate, DiaRoot BCS differs by not containing aluminum, which may influence its sealing properties and performance. Biocera, another recent addition to BCS, is supplied in a pre-mixed injectable paste system. While the physical properties of Biocera are not yet fully explored in the literature, its composition is stated by the manufacturer to include calcium aluminate, calcium silicate, calcium hydroxide, accelerators and zirconium oxide, along with some thickening agents. The teeth that were obturated with Biocera BCS showed more leakage compared to all other sealers used in this study. Interestingly, Jasrotia *et al.* [23] reported a statistically significant difference in dye penetration between Ceraseal BC (5.32 mm) and AH Plus (8.06 mm), but these results may vary due to the difference in the study design. Jasrotia *et al.* used a broader selection of single-rooted permanent teeth, while this study specifically focused on single-rooted mandibular premolars. Kaul *et al.* [9], in their study, compared the conventional and ceramic-coated bioceramic sealers with GuttaFlow and AH Plus sealers and found that both the bioceramic sealers had a mean dye penetration of 0.89mm while GuttaFlow and AH Plus had 1.42 and 1.73 mm, respectively. Although these results suggested that bioceramics have better sealing ability, the results were not statistically significant. Similarly, Haji *et al.* [24] compared the sealing ability of Ceraseal with Zinc Oxide Eugenol and documented that Ceraseal exhibited superior sealing. Additionally, Kunam *et al.* [25] found that bioceramic sealers demonstrated superior wettability compared to AH Plus, which could further enhance their sealing properties by improving the bond-interaction between the sealer and the RC walls. These studies, along with this study's findings, suggest that bioceramic sealers may offer promising alternatives to traditional resin-based sealers. Although some studies, such as those by Mert and Gencoglu [21], assessed the physical properties of DiaRoot and Lee *et al.* [26] evaluated its bond strength and intratubular biomineralization, limited research is available on its sealing ability. This underscores the need for further studies to evaluate the sealing performance of DiaRoot BC and other newer bioceramic sealers. Dye penetration, while an effective method for assessing microleakage *in vitro*, does not necessarily reflect the material's ability to prevent bacterial infiltration in a living system. Therefore, *in vivo* studies are needed to evaluate how such materials interact and perform in a clinical setting, where biological factors may influence the sealing performance. Advanced techniques such as CLSM, SEM and transmission electron microscopy could provide more detailed insights into the surface topography and chemical interactions of these sealers, which would complement the findings from dye penetration studies [27]. Moreover, clinical investigations would help to establish the long-term outcomes of BCS, such as their capacity to withstand the forces encountered in the oral cavity over an extended period and their potential for reducing treatment failures. Understanding these factors will be crucial for determining whether BCS, including CeraSeal BCS, can truly serve as viable alternatives to traditional RBS like AH Plus.

## Conclusion:

We show that there are significant variations in the sealing potential of the four sealers. Ceraseal BCS exhibited the highest sealing ability among all, followed by AH plus. It was superior to the DiaRoot and Biocera sealers.
